# Introductory

**Published:** 1890-01

**Authors:** 


### HOMŒOPATHIC PHYSICIAN,

A MONTHLY JOURNAL OF

HOMŒOPATHIC MATERIA MEDICA AND CLINICAL MEDICINE.

If our school ever give up the strict inductive method of Hahnemann, we are lost, and deserve only to be mentioned as a caricature in the history of medicine."—CONSTANTINE HERING.

Vol. X. JANUARY, 1890. No. 1.

### INTRODUCTORY.

As was announced in the last number of this journal, the title-page will show a change in editors. Although there has been a change in editors, there will be no change in the principles which have been adhered to since the journal was started. It is our intention to still bear aloft the best that is known and thought of Hahnemann's Homœopathy. To this end we invite the co-operation of the followers of Hahnemann. It must be borne in mind that the editors alone cannot make an interesting and successful medical journal. And we wish to have all friends of the cause remember that our work is done with the sole aim of advancing the good work of genuine Homœopathy, and the only compensation we expect is the knowledge that we have attempted and succeeded in doing this. We propose to be aggressive. We feel competent to defend our cause, having good reason for the faith that is in us. We shall strike a blow for our principles whenever and wherever we think necessary. Now, as heretofore, let all who are in our ranks assist us in the fight, and we shall shrink from nothing that we think right. In the line of duty we are without fear, and we trust we shall be without reproach. G. H. C.

